# Polyphenol from *Rosa* *roxburghii* Tratt Fruit Ameliorates the Symptoms of Diabetes by Activating the P13K/AKT Insulin Pathway in db/db Mice

**DOI:** 10.3390/foods11050636

**Published:** 2022-02-22

**Authors:** Chao Chen, Shuming Tan, Tingyuan Ren, Hua Wang, Xiaotong Dai, Hui Wang

**Affiliations:** 1 Key Laboratory of Agricultural and Animal Products Storage and Processing of Guizhou Province, College of Liquor and Food Engineering, Guizhou University, Guiyang 550025, China; chaochengzu@163.com (C.C.); tyren@gzu.edu.cn (T.R.); wh978280094@163.com (H.W.); ogcogcogcogc@163.com (X.D.); 2 Institute of Biotechnology, Guizhou Academy of Agricultural Sciences, Guiyang 550025, China; wanghui880101@163.com

**Keywords:** type 2 diabetes, fasting blood glucose, hypoglycemic, oxidative stress, FOXO1

## Abstract

About 4% of the world’s population has type 2 diabetes mellitus (T2DM), and the available hypoglycemic drugs for treating diabetes have some side effects. Therefore, research on the extraction of hypoglycemic components from plants has gradually become popular. This study aimed to investigate the hypoglycemic effects of polyphenol-rich *Rosa roxburghii* Tratt extract (RP) isolated from *Rosa roxburghii* Tratt fruit and of four constituents (IRP 1–4 ) isolated from RP on db/db mice. The results indicated that the oral administration of RP and IRP 1–4 could markedly decrease the food intake, water intake, fasting blood glucose (FBG), and serum insulin levels in the db/db mice. Glucose intolerance, insulin resistance, and oxidative stress were ameliorated in the RP and IRP 1–4 groups. Histopathological observation revealed that RP and IRP 1–4 could effectively protect the liver fat against damage and dysfunction. RP and IRP 1–4 also increased the hepatic and muscle glycogen contents by increasing the phosphorylation and reducing the expression of glycogen synthase kinase 3β (GSK3β). The activities of glucokinase (GCK), phosphoenolpyruvate carboxylase (PEPCK), and glucose-6-phosphatase (G6PC) and their respective mRNA expression levels in the liver of db/db mice were simultaneously increased and decreased in the intervention groups. RP and IRP 1–4 significantly increased the expression of phosphatidylinositol 3-kinase (P13K) and the phosphorylation of protein kinase B (AKT). These results indicate that RP and IRP 1–4 exhibit good hypoglycemic effects by activating the P13K/AKT signaling pathway and regulating the expression of FOXO1 and p-GSK3β proteins, controlling hepatic gluconeogenesis and improving hepatic glycogen storage insulin resistance. Therefore, RP and IRP 1–4 could be utilized as the hypoglycemic functional component to alleviate the symptoms of T2DM.

## 1. Introduction

Type 2 diabetes mellitus (T2DM) is a systemic metabolic disorder disease. Currently, approximately 4% of the global population lives with diabetes, and therefore it is a worldwide problem. The prevalence of T2DM is directly proportional to age, body mass index, fasting blood glucose (FBG), and blood pressure [1]. Long-term hyperglycemia in T2DM patients can lead to cardiovascular disease, nephropathy, retinopathy, and other complications. T2DM is triggered by insulin resistance (IR), caused by decreased glucose uptake and insulin sensitivity due to obesity, ageing, and sedentary lifestyle [2]. IR manifests as increased gluconeogenesis, decreased hepatic glycogen content, and abnormal insulin signaling. Numerous studies have revealed that the key enzymes of glucose metabolism are glucokinase (GCK), glucose-6-phosphate kinase (G6PC), and phosphoenolpyruvate carboxylase (PEPCK). Decreased GCK activity and increased G6PC and PEPCK activities are the leading causes of T2DM [3]. The phosphatidylinositol 3-kinase/protein kinase B (P13K/AKT) pathway is one of the major insulin signaling pathways focused on in the current research; the abnormal expression of factors associated with this pathway are some of the primary causes of T2DM. Some hypoglycemic drugs, such as metformin and repaglinide, can cause nausea and vomiting. The side effects of these drugs can be reduced, and natural plant-derived hypoglycemic ingredients can increase their safety as an adjunct to drug therapy [4].

*Rosa roxburghii* Tratt fruit (RRT) is a perennial deciduous shrub of the family Rosaceae, mainly distributed in the southwestern regions of China, especially in Guizhou province. RRT is an edible wild fruit with a slightly sour astringent flavor, strong aroma, and crunchy texture. RRT is planted in approximately 1173 square kilometers across Guizhou province, and its annual output is 130,000 tons [5]. According to the records of the “Compendium of Materia Medica”, throughout history, RRT has been used as a traditional Chinese medicine to intervene with diseases [6]. RRT is known as “the King of Vitamin C”, and according to previous reports, RRT contains 1300–3500 mg of vitamin C per 100 g fresh weight, much higher than any fruit. In addition, RRT is rich in polyphenolic compounds, which have antioxidant, hypoglycemic, and anti-inflammatory properties. The total phenolic content of RRT is 108.9–156.3 mg GAE per g dry weight, again much higher than other fruit. Phenolic compounds primarily include ellagic acid, quercitrin, and chlorogenic acid [7]. Polyphenols are secondary plant metabolites, which can help improve carbohydrate metabolism, glucose tolerance, and IR, and can elevate muscle and liver glycogen contents [8]. Lenh Vo Van et al. discovered that the extract of *Merremia tridentta*, which is rich in quercitrin, exhibited strong α-amylase and α-glucosidase inhibitory activities [9]. Luo et al. illustrated that polyphenol-rich extract from sweet potato leaves could lower FBG, improve glucose tolerance and liver glycogen content, and up-regulate the P13K/AKT/glucose transporter 4 signaling pathway, thereby promoting glucose transport [10].

We used the ultrasound-assisted combined cellulase method to extract RP and total polysaccharide RRT extracts and explored their effects on glucose and lipid metabolism disorders in T2DM mice. The results revealed that RP could improve T2DM with better results than polysaccharide extract [11]. In the current research, we investigated the hypoglycemic activity and potential mechanisms of RP and IRP 1–4 in db/db mice. The current work will present new insights into the prevention and treatment of T2DM by RP.

## 2. Materials and Methods

### 2.1. Reagents

RRT (NO.5 GuiNong, picked in early September 2021, stored at −80 °C; the RRT used in the experiment were the same type and the same batch) were purchased from Guizhou Hongcai Investment Company (Guizhou, China). Metformin hydrochloride (purity 97%), chlorogenic acid (purity 98%), quercitrin (purity 98%), and ellagic acid (purity 98%) were purchased from Shanghai Maclean Biochemical Technology Co., Ltd. (Shanghai, China). Total superoxide dismutase (T-SOD), total antioxidant capacity (T-AOC), catalase (CAT), and malondialdehyde (MDA) kits were purchased from Nanjing Jiancheng Biological Engineering Institute (Nanjing, China). Total protein quantification (BCA), liver glycogen, muscle glycogen, glucokinase (GCK), glucose-6-phosphate kinase (G6PC), insulin, and phosphoenolpyruvate carboxykinase (PEPCK) assay kits were purchased from Fujian Quanzhou Ruixin Biotechnology Co., Ltd. (Quanzhou, China).

### 2.2. Sample Preparation

RP was obtained through previous preparation in our laboratory (briefly, RRT fruits were freshly harvested, transported to the laboratory using a cold chain where they were vacuum dried in a freezing environment and then grounded into powder form. We used 70% ethanol and 0.25% cellulase as the extraction solvent, and the active substances were extracted through an ultrasound-assisted method. RRT extracts were purified using AB-8 macroporous resin and then extracted with ethyl acetate to obtain RP, whereas 4 of its constituents were separated using TBE-300B high-speed counter-current chromatography (Shanghai Tongtian Biological Co., Ltd.; Shanghai, China). Four components were named IRP-1, IRP-2, IRP-3, IRP-4. Analysis of the 4 constituents was performed using high-performance liquid chromatography (HPLC) according to the method by Gao et al. with slight modifications [12]. Chromatographic columns: ZORBAX SB-C18 column (4.6 × 250 mm^2^, 5 μm). Chromatographic conditions: mobile phase (A: 0.05% formic acid, B: methanol), gradient elution: 0–10 min, 10% B; 10–25 min, 25% B; 25–35 min, 40% B; 35–55 min, 80% B; 55–65 min, 10% B. Flow rate: 1.0 mL/min. Sampling volume: 10 μL. Column temperature: 25 °C The absorbance of samples was monitored at 254 nm. Peaks were identified by comparing the retention time in a specific wavelength. Mass spectrometry conditions: heated electrospray ionization source, detection in negative ion mode, spray voltage 3.2 kV, capillary temperature 320 °C, sheath gas (N_2_) flow rate 10 arb. Scanning mode was primary mass spectrometry, with a scanning range of m/z 100~1500.

### 2.3. Animal Experiments

Male C57/BKS-db/db and C57/BKS db/m mice at 4–5 weeks of age (Gem Pharmatech Co., Ltd., Nanjing, China) were approved by the Animal Experiment Ethics Committee Guizhou University (number: EAE-GZU-2020-P010). Fifty-four mice (*n* = 6 per group) were housed in a well-ventilated room at 23 ± 2 °C with relative humidity between 45% and 65% and exposed to 12/12 h of alternating light and dark environments. The mice were allowed to eat and drink freely. After 1 week of the adaptation period, db/m mice were assigned as the control group, and db/db mice were randomly divided into 8 intervention groups, including the model group, RP-100 group, RP-200 group, IRP-1 group, IRP-2 group, IRP-3 group, and IRP-4 group. The dose of each experimental group was 50 mg/kg, except for RP-100 and RP-200 groups. The doses of RP-100 and RP-200 groups were 100 mg/kg and 200 mg/kg, respectively. Positive (metformin hydrochloride) and samples were converted to net weight according to purity and dissolved in sterile saline to prepare the solution, respectively. The dose of oral administration was adjusted according to the bodyweight of the mice, and the dose was given at the same time every day. The control and model groups were given normal saline solutions orally. The intervention period was 8 weeks. Food and water consumed, FBG, and body weight were recorded once a week. On the last day, after 12 h of fasting without water, a blood sample was taken from the eyes and stored in a centrifuge tube. The blood was centrifuged at 3500× *g* at 4 °C for 15 min, and the upper serum was aspirated and stored at −80 °C for measurement. The mice were dissected, and the liver and skeletal muscle were cleaned with saline solution and divided into 5 parts, of which 4 parts were stored at −80 °C and 1 part in formaldehyde fixative for testing.

### 2.4. Determination of Serum Insulin Concentration

According to the manufacturer’s instructions, the serum insulin concentration was determined using an enzyme-linked immunosorbent assay (ELISA) kit (Fujian Quanzhou Ruixin Biotechnology Co., Ltd., Quanzhou, China). The serum insulin resistance index (HOMA-IR) was calculated according to the following formula: HOMA-IR = fasting serum glucose (mmol/L) × fasting serum insulin (mU/L)/22.5.

### 2.5. Oral Glucose Tolerance Test

In the seventh week, mice were fasted for 12 h and orally administered 2.00 g/kg BW of glucose. Then, their blood was taken by clipping the tails to measure blood glucose levels at 0 h, 0.5 h, 1 h and 2 h. The area under the blood glucose curve (AUC) was calculated according to the following formula: AUC (hmmol/L) = 0.5A + B + C + 0.5D/2. In the formula, A, B, C and D represent the blood glucose values at 0 h, 0.5 h, 1 h and 2 h, respectively, after oral gavage of glucose solution.

### 2.6. Determination of Antioxidant Enzyme Activities

The kit was used according to the manufacturer’s instructions (Nanjing Jiancheng Biotechnology Institute China; Nanjing, China). MDA contents in serum and liver were measured using the TBA method, and the T-SOD activities in serum and liver were measured using the hydroxylamine method. T-AOC capacity and CAT content in the liver were measured using the FRAP and molybdenum acid methods.

### 2.7. Measurement of Hepatic Glycogen Content and Muscle Glycogen Content

The glycogen concentrations were determined using a commercial assay kit (Fujian Quanzhou Ruixin Biotechnology Co., Ltd., China). In brief, liver and muscle tissues were prepared using 1% liver glycogen assay solution and 5% muscle glycogen assay solution. The glycogen concentrations were tested after adding the detection reagent and measured at 620 nm using a UV spectrophotometer. Glycogen contents are expressed as mg/g tissue.

### 2.8. Histopathological Examination

The fixed livers were trimmed, dehydrated, embedded, sectioned, stained, sealed, and then observed under a microscope, and images were taken.

### 2.9. Activities of GCK, G6PC and PEPCK

GCK, G6PC, and PEPCK activities were determined using a commercial assay kit (Fujian Quanzhou Ruixin Biotechnology Co., Ltd., China) according to the manufacturers’ instructions. The activities were expressed as mU/mg and U/g, respectively.

### 2.10. RNA Extraction and Quantitative Real-Time Polymerase Chain Reaction

The total RNA extraction procedure was performed as follows. Briefly, 100 mg of tissue was grounded thoroughly until no visible tissue clumps were present. The supernatant was extracted through centrifugation at 12,000× *g*. Then, 250 μL of trichloromethane was added, and the tube was shaken upside down for 15 s to mix it thoroughly, followed by keeping it stable for 3 min. The tube was centrifuged at 12,000 rpm for 10 min at 4 °C. Finally, 400 μL of supernatant was transferred to a new tube, followed by adding 0.8 times the volume of isopropanol, and the tube was kept stable for 15 min at −20 °C. Finally, the tube was centrifuged at 12,000× *g* for 10 min at 4 °C, and the white precipitate at the bottom of the tube was collected as the RNA. Then, 15 μL of RNA-free water was used to dissolve the RNA and incubated at 55 °C for 5 min. The reverse transcription system preparation and program settings are shown in Tables S1 and S2. Quantitative PCR steps: 0.2 mL PCR tubes were taken, and the reaction system was prepared as mentioned in Table S3. Three tubes were prepared for each reverse transcription product. The PCR amplification method is shown in Table S4. Table 1 lists the sequences of the primers. The relative amounts of all mRNAs were calculated using the comparative cycle threshold (Ct) method, with the formula 2^−ΔΔCt^; *GADPH* was used as an internal control.

### 2.11. Western Blot Analysis

Briefly, tissues were cut into fine pieces, and the total protein was extracted using a lysis buffer. The protein concentration was determined using the BCA method, and equal amounts of protein were denatured and separated by SDS-PAGE electrophoresis in the ratios shown in (Table S5). After the termination of electrophoresis, proteins were transferred to polyvinylidene difluoride membranes. Then, skim milk was added to close the membrane for 30 min and P13K, FOXO1, p-AKT and total AKT, p-GSK3β and total GSK3β were detected using primary antibodies. Then, the membranes were incubated with secondary antibodies for 30 min (1:5000 dilution). After a full reaction with high sensitivity ECL solution in a dark room, the membrane was developed and fixed with reagents. GADPH was used as a loading control.

### 2.12. Statistical Analysis

Statistical analyses in the current study were performed using SPSS 22.0, and the measurement data were expressed as mean ± standard deviation (x ± s). The Duncan method was used for two-way comparisons between groups, and differences were expressed at *p* < 0.05, which denoted statistical significance.

## 3. Results

### 3.1. Analysis of Phenolic Profiles in RRT

**Figure 1:**
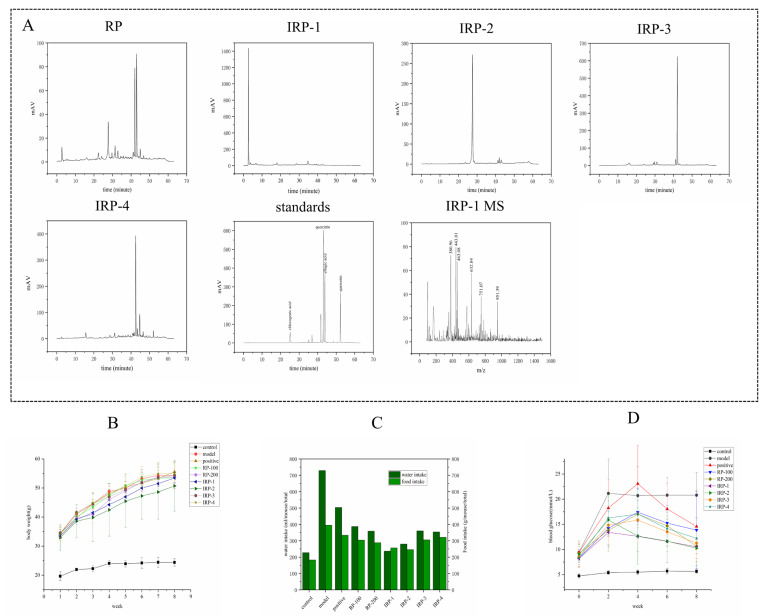
HPLC analysis of RP, IRP 1–4, standard reference samples, and effects of RP and IRP 1–4 on weight, food and water intake, FBG in db/db mice of 0 and 8 weeks. (**A**) HPLC of total phenolic compounds (RP) in RRT and the 4 main components isolated from RP. Effects of RP and IRP 1–4 on the body weight (**B**), food intake and water intake (**C**), and fasting blood glucose (**D**) in the db/db mice. All data are presented as the means ± SD (*n* = 6) except food and water intake.

As seen in Figure 1A, RP contained four phenolic constituents, which were identified by comparing them with the authentic samples. IRP 2–4 could be chlorogenic acid, quercitrin, and ellagic acid, respectively. High-performance liquid chromatography coupled with mass spectrometry revealed that IRP-1 produced a molecular ion peak with [M-H] of 951. Therefore, we inferred that the molecular weight of IRP-1 was 951. IRP-1 has the same molecular weight as ellagitannin (praecoxin A), and ellagic acid is usually derived from ellagitannins, so IRP-1 might be an ellagitannin. Ellagitannins have significant antioxidant, anticancer, and antitumor activities [13]. Kun Dai et al. demonstrated that chlorogenic acid in peaches has significant antioxidant and hypoglycemic effects, which could inhibit α-glucosidase, α-amylase and scavenges free radical [14]. Aphichat et al. discovered that quercetin could improve glucose metabolism and glucose tolerance [15]. Yin et al. revealed that ellagic acid-rich oak extracts notably reduced serum MDA and SOD and inhibited α-glucosidase and α-amylase activities in diabetic mice [16]. So, we studied the effects of RP and IRP 1–4 on glucose metabolism and identified the underlying mechanism using db/db mice.

### 3.2. Effects of RP and IRP 1–4 on Bodyweight, Food Intake, Water Intake and FBG

As shown in Figure 1B, compared with the control group, the weight of the model group increased rapidly and decreased in the seventh week. The trend of weight gain in the IRP-1 and IRP-2 groups slowed down compared with the model group. Experimental groups did not exhibit any weight loss during the intervention period except the model group. As shown in Figure 1C, compared with the control group, the food and water intake of the model group were significantly higher. After 8 weeks of intervention, the food and water intake of each intervention group decreased significantly. Among them, IRP-1 and IRP-2 groups exhibited the most significant effects. As shown in Figure 1D, the FBG control group remained stable during the intervention period, and that of the model group remained stable after a substantial increase in the first 2 weeks. The rising trend of FBG in each intervention group declined in the first 2 weeks and decreased significantly after week 4, and the IRP-1 and IRP-2 groups exhibited the most significant effects.

### 3.3. Effects of RP and IRP 1–4 on Glucose Tolerance, Insulin Concentrations and HOMA-IR

**Figure 2:**
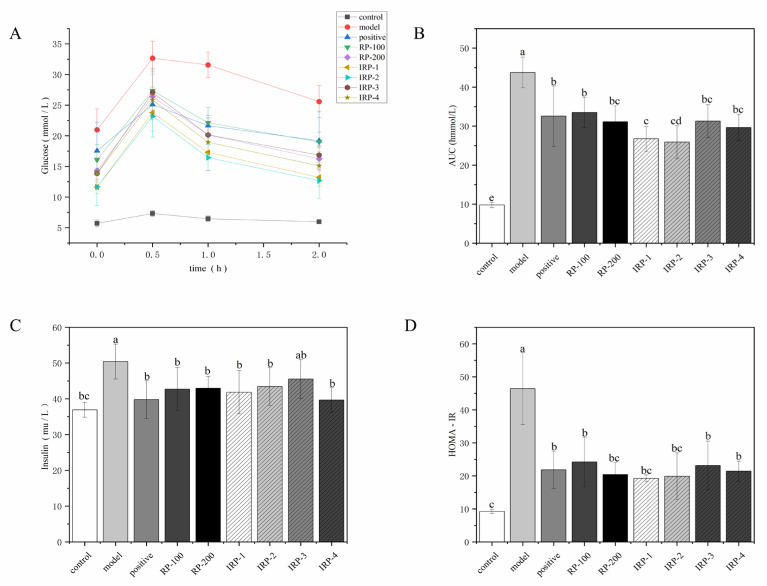
Effects of RP and IRP 1–4 on glucose intolerance and ameliorated insulin resistance in db/db mice. (**A**) oral glucose tolerance test, (**B**) AUC of glucose, (**C**) fasting serum insulin concentrations, (**D**) HOMA-IR. Data are presented as mean ± SD (*n* = 6). Different letters (superscripts a–e) are significantly different at *p* < 0.05.

As shown in Figure 2A, compared with the model group, the glucose tolerance of the mice in each intervention group was significantly improved. In the first 30 min, the glucose levels in all groups rose rapidly and reached a peak at 30 min, then began to decline slowly. IRP-1 and IRP-2 groups demonstrated the fastest downward trends among the intervention group. As shown in Figure 2B, compared with the control group, the AUC of the model group was markedly increased (*p* < 0.05). Compared with the model group, the AUC of each intervention group was significantly decreased (*p* < 0.05). The AUC of the IRP-1 and IRP-2 groups were significantly decreased compared with other intervention groups (*p* < 0.05). The positive, RP-200, IRP-3, and IRP-4 groups exhibited similar effects. As shown in Figure 2C, compared with the control group, the serum insulin concentration of the mice in the model group was significantly increased (*p* < 0.05). After 8 weeks of intervention, the serum insulin concentration in each intervention group decreased significantly (*p* < 0.05). The IRP-3 group exhibited no significant difference (*p* > 0.05) compared with the model group. The positive and IRP-4 groups demonstrated the most significant effects (*p* < 0.05), decreasing by 21.12 and 21.32%, respectively. As shown in Figure 2D, compared with the control group, the model group had severe insulin resistance and increased HOMA-IR (*p* < 0.05). After 8 weeks of intervention, the HOMA-IR of each intervention group was significantly decreased (*p* < 0.05). The IRP-1 and IRP-2 groups represented the most significant effects.

### 3.4. Effects of RP and RP 1–4 on Oxidative Stress

As shown in Table 2, compared with the control group, the MDA contents in the serum and liver of the model group increased significantly by 126.43% and 118.21%. After 8 weeks of intervention, the MDA content of each intervention group decreased significantly compared with the model group (*p* < 0.05). The serum MDA content of RP-200, IRP-1 and IRP-4 groups differed significantly from the other intervention groups (*p* < 0.05). The differences in liver MDA contents in intervention groups were insignificant (*p* > 0.05). Compared with the control group, the T-SOD activities in serum and liver of the model group decreased significantly (*p* < 0.05). After 8 weeks of intervention, the T-SOD activity of each intervention group increased significantly (*p* < 0.05), except for the RP-100 group. The difference between the serum T-SOD activities of the IRP-1 and control groups were not significant (*p* > 0.05), and the T-SOD activity of each intervention group difference was not significant (*p* > 0.05). Compared with the control group, the T-AOC levels and CAT activity in the model group were significantly decreased (*p* < 0.05). The T-AOC levels and CAT activity in each intervention group were significantly proved. The T-AOC levels and CAT activity in the positive and RP-100 groups were not significantly different from the model group (*p* > 0.05). These results indicate that RP and RP 1–4 could improve the oxidative stress phenomenon and the antioxidant ability of the liver in the db/db mice. Overall, the RP-200, IRP-1, and IRP-2 groups exhibited the most remarkable effects.

### 3.5. Effects of RP and IRP 1–4 on Micromorphology of Liver

**Figure 3:**
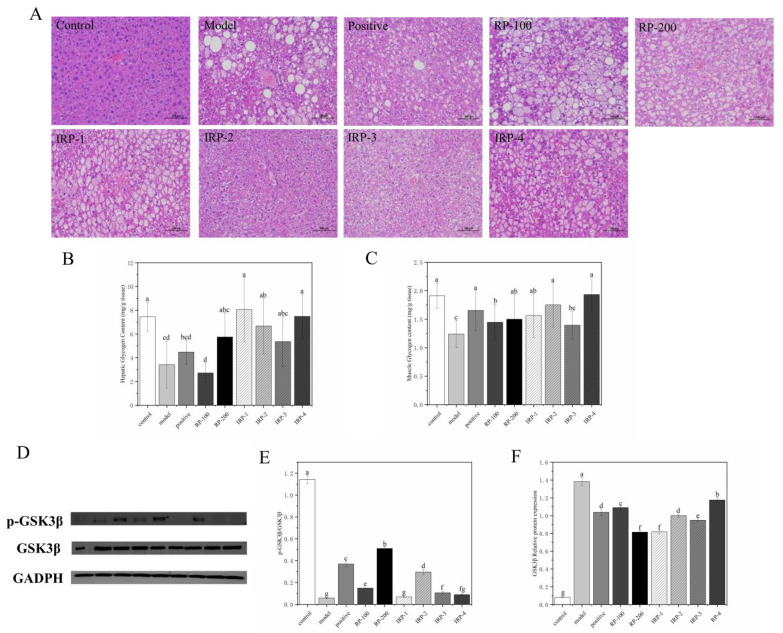
Effects of RP and IRP 1–4 on Micromorphology of Liver, hepatic glycogen content, muscle glycogen content, and GSK3β protein expression in db/db mice. (**A**) H&E staining of the liver (×200 magnification), (**B**) hepatic glycogen content, (**C**) muscle glycogen content. (**D**) Representative Western blots depict p-GSK3β, GSK3β, and GADPH. (**E**) RP and IRP 1–4 decreased the GSK3β protein level. (**F**) RP and IRP 1–4 increased p-GSK3β/GSK3β. All data were expressed as mean ± SD (*n* = 6). Different letters indicate a significant difference (*p* < 0.05).

As shown in Figure 3A, the hepatocyte in the control group was uniform in size, neatly arranged, and no fatty degeneration or oedema were observed. In the model group, hepatocyte was disordered, with numerous intracellular fat vacuoles and severe visible hepatocyte degeneration. After 8 weeks of intervention, the morphological structure of hepatocytes, fat cell degeneration, ballooning, and fat vacuolation were significantly improved in each intervention group. The improvement effect was weak in the RP-100 group, and the improvement effects of hepatocyte morphology were more satisfactory in the IRP-2 and IRP-4 groups.

### 3.6. Effects of BRD on Hepatic Glycogen Content, Muscle Glycogen Content and Expression of Glycogen Synthesis-Related Proteins

As shown in Figure 3B,C, the contents of liver and muscle glycogen in mice were measured using the ELISA method, and the key enzymes of glycogen synthesis were detected using the WB method. Compared with the control group, the liver and muscle glycogen contents of the model group were significantly reduced by 54.24% and 35.16%, respectively (*p* < 0.05). After the intervention, glycogen content increased in all groups except the RP-100 group. The liver glycogen contents of IRP-1, IRP-2, and IRP-4 groups significantly increased by 148.09%, 95.89% and 119.65%, respectively, compared with the model group (*p* < 0.05). The muscle glycogen contents of all intervention groups were not significantly different from the control group except the RP-100 and IRP-3 groups (*p* > 0.05).

The activated phosphorylation of GSK3β protein can promote glycogen synthesis. As shown in Figure 3D–F, compared with the control group, the expression of GSK3β protein was significantly increased (*p* < 0.05). After the intervention, positive and all samples could significantly reduce the expression of GSK3β protein. The RP-200 and IRP-1 exhibited the most significant effects. In addition, the p-GSK3β/GSK3β ratio of the RP-200 and IRP-2 groups significantly increased compared with the model group (*p* < 0.05). These results indicate that IRP-2 is the principal active substance that increases GSK3β protein phosphorylation in RP.

### 3.7. Effects of RP and IRP 1–4 on Activities of GCK, G6PC, PEPCK and the Expression of the Gluconeogenesis-Related Gene

**Figure 4:**
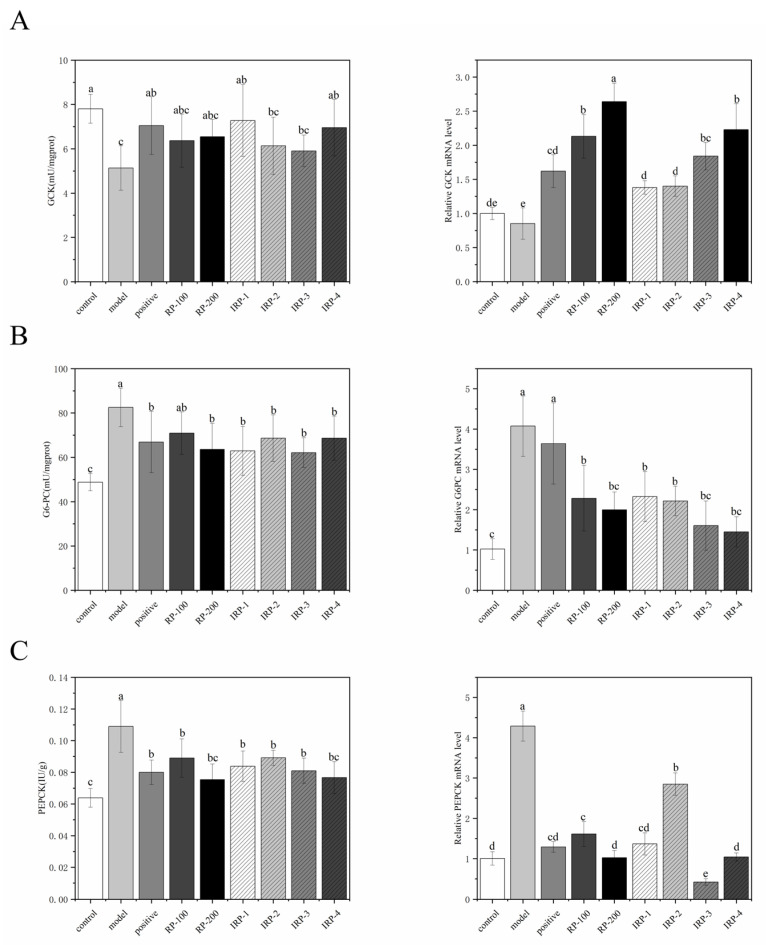
Effects of RP and IRP 1–4 on hepatic gluconeogenesis in T2DM mice. RP and IRP 1–4 increased the activities of *GCK* (**A**) and decreased the activities of *G6PC* (**B**) and *PEPCK* (**C**). RP and IRP 1–4 increased the relative mRNA levels of *GCK* (**A**) and reduced the relative mRNA levels of *G6PC* (**B**), and *PEPCK* (**C**). All data were expressed as the means ± SD (*n* = 6). Different letters indicate a significant difference (*p* < 0.05).

As shown in Figure 4A, GCK activity in the model group decreased significantly by 34.62% (*p* < 0.05) compared with the control group. After the intervention, GCK activities in the positive, IRP-1, and IRP-4 groups increased significantly by 38.04%, 41.72% and 35.48% (*p* < 0.05) more than the model group, respectively. GCK activity differences among the three groups were not significant (*p* > 0.05). Compared with the model group, GCK activities in the RP-100, RP-200, IRP-2 and IRP-3 groups increased by 24.17%, 27.49%, 19.49% and 15.01%, respectively, but the difference was not significant (*p* > 0.05). As seen in Figure 4B,C, compared with the control group, G6PC and PEPCK activities in the model group were significantly increased by 69% and 70.31%, respectively (*p* < 0.05). After the intervention, G6PC activities decreased significantly in all groups compared with the model group except for the RP-100 group (*p* < 0.05). G6PC activity in the IRP-3 group decreased by 24.75%, the most significant of all. PEPCK activities were significantly reduced in all intervention groups compared with the model group (*p* < 0.05). PEPCK activities in RP-200 and IRP-4 groups decreased by 31.19% and 29.64%, respectively, which were not significantly different from the control group (*p* > 0.05).

The mRNA expression of *GCK*, *G6PC,* and *PEPCK* was measured using qPCR to verify their enzymatic activities. *GCK* mRNA expression was significantly increased in each intervention group compared with the model group, including a 214.12% and 162.35% increase in the RP-200 and IRP-4 groups, respectively (*p* < 0.05). The mRNA expressions of *G6PC* and *PEPCK* were significantly decreased in all intervention groups (*p* < 0.05). *G6PC* mRNA expressions decreased by 50.86%, 60.44%, and 64.37% in the RP-200, IRP-3, and IRP-4 groups, respectively, not significantly different from the control group (*p* > 0.05). The mRNA expressions of *PEPCK* in the RP-100 and IRP-2 groups were significantly different from the control group (*p* < 0.05), whereas the remaining intervention groups were not significantly different from the control group (*p* > 0.05).

### 3.8. Effects of RP and IRP 1–4 on P13K/AKT Insulin Signaling Pathway

**Figure 5:**
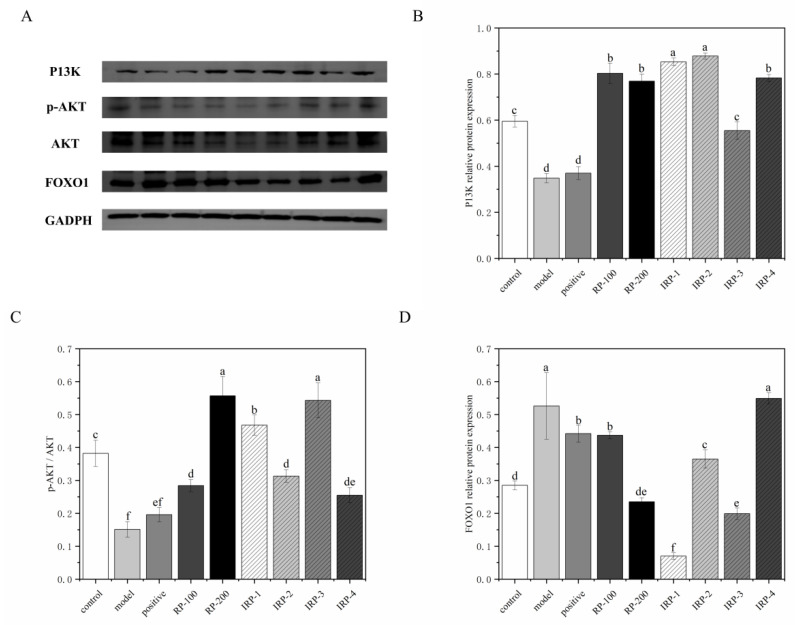
Effects of RP and IRP 1–4 on the PI3K/AKT/FOXO1 insulin signaling pathway in db/db mice. (**A**) Representative Western blots depicting P13K, p-AKT and total AKT, FOXO1, and GADPH. (**B**–**D**) RP and IRP 1–4 increased the relative protein expression of P13K (B) RP, and IRP 1–4 increased p-AKT/AKT (**C**) RP and IRP 1–4 decreased the relative protein expression of FOXO1 (**D**). All of the samples were evaluated in triplicate. All data are presented as mean ± SD (*n* = 6). Different letters indicate a significant difference (*p* < 0.05).

The P13K/AKT signaling pathway is a key signaling pathway that inhibits IR. FOXO1 is a downstream protein of the P13K/AKT pathway, and excessive activation of FOXO1 protein leads to insulin failure leading to oxidative stress and IR in the body [17]. As shown in Figure 5B–D, compared with the control group, the protein level of P13K and the ratio of p-AKT/AKT were significantly decreased in the model and positive groups (*p* < 0.05). Moreover, the protein level of FOXO1 was significantly increased in the model group (*p* < 0.05). After the intervention, the protein level of P13K and the p-AKT/AKT ratio were significantly increased in all intervention groups, except in the positive group (*p* < 0.05). The protein level of FOXO1 was markedly decreased in all intervention groups, except in the IRP-4 group. IRP-1 demonstrated the most significant effect, and IRP-4 exhibited a general effect.

## 4. Discussion

C57 BLKS/JGPT db/db mice are characterized by IR and hyperglycemia due to their knocked-out leptin receptors, and their external insulin cannot control the levels of blood glucose and hepatic gluconeogenesis. Db/db mice were used as a model for T2DM research; we successfully separated the four polyphenols from RP by HSCCC and studied the hypoglycemic activity and pathways of action of RP and IRP 1–4.

During the 8 weeks of the intervention period. The mice in the model group revealed a significant increase in body weight and started to lose weight at the 7th week, with a significant increase in food and water intake and FBG. These phenomena indicated that the mice had suffered from severe T2DM. The water intake, food intake, and FBG of the mice in each intervention group were significantly reduced, indicating that the T2DM of the mice was significantly improved. The IRP-1 and IRP-2 groups exhibited the most significant effects. These outcomes are consistent with the studies of Andrea Da et al., who suggested that carbohydrate metabolism and reduced intake could be improved by polyphenols-rich foods [8].

Glucose tolerance refers to the body’s ability to regulate blood glucose concentration. Severe obesity and the long-term ballooning of hepatocytes can reduce glucose tolerance [18]. The OGTT capacity of the mice in the model group was severely reduced, and the AUC was significantly increased (*p* < 0.05). Both drugs and IRP 1–4 significantly enhanced the glucose tolerance of the db/db mice. This is consistent with the result of Dejing Chen et al., who suggested that polyphenols in *Camellia sinensis* var (Chinese tea) could improve OGTT in T2DM mice [19]. Insulin is a peptide hormone consisting of 51 amino acids. It is an essential hormone regulating glucose, protein, and fat energy metabolism. Decreased insulin sensitivity and the reduction in the insulin-mediated inhibition of gluconeogenesis are the primary causes of T2DM [20]. The serum insulin level and HOMA-IR of mice in the model group were significantly increased (*p* < 0.05). The serum insulin levels and HOMA-IR of mice in each intervention group were significantly decreased. IRP-1 and IRP-4 groups demonstrated the most significant effects. These results indicate that RP and IRP 1–4 increased insulin sensitivity, reducing serum insulin secretion and improving IR. This agrees with Gary Williamson et al., who suggested that polyphenols could be used as supplements to improve IR, lower postprandial glucose, and regulate glucose transport [21].

Oxidative stress is one of the primary mechanisms which causes T2DM. An antioxidant system consists of active antioxidant enzymes in biological cells, such as CAT and SOD. When the rate of free radical production exceeds the antioxidant defense system, numerous toxic products (MDA) accumulate, resulting in oxidative stress [22]. Oxidative stress damages islet and liver cells through lipid peroxidation, mitochondrial dysfunction, and DNA damage, leading to T2DM [23]. MDA content was significantly increased, and T-AOC, CAT, and SOD activities were significantly decreased in the model mice (*p* < 0.05). These results indicate that oxidative stress was severe in T2DM mice. After the intervention, MDA content was significantly decreased, and T-AOC, SOD, and CAT activities were markedly increased in each intervention group. RP-200, IRP-1, IRP-2 and IRP-4 had the most significant effects. These results indicate that RP and IRP 1–4 improved oxidative stress and repaired cells in T2DM mice. The effects of IRP 1–4 were superior to RP, and RP’s effect was positively correlated with the dose effect. These results are consistent with Sajad Fakhri et al., who suggested that the upstream dysregulated oxidative stress signaling pathway can be regulated, and plant polyphenols can improve oxidative stress [24].

Pathological phenomena, such as lipid vacuolization, cell ballooning, massive degeneration of adipocytes, and oedema, were demonstrated in the livers of T2DM mice. The pathological damage was severe in the model group mice. The pathological damage was improved in all intervention groups, with the most significant improvement in the IRP-2 and IRP-4 groups. These are consistent with the results of Rong Li et al., who noted that pathological damage of pancreatic islet cells and liver cells can be improved by cinnamon polyphenols in T2DM mice [25]. The glycogen storage in the liver and skeletal muscle, promoted by insulin, is indispensable for maintaining glucose homeostasis. Increased GSK3β protein expression and decreased GSK3β protein phosphorylation are often observed in T2DM animals with insulin resistance [26]. GSK3β protein negatively affects the insulin signaling pathway and phosphorylates various substrates, leading to elevated blood glucose [27]. The model group markedly reduced liver glycogen and muscle glycogen contents (*p* < 0.05). The glycogen content was significantly elevated in all intervention groups except the RP-100 group. IRP-1, IRP-2 and IRP-4 were significantly more effective than IRP-3.

The current study demonstrated that three ellagitannins and ellagic acid exhibited excellent hypoglycemic effects [28]. The phosphorylation of GSK3β could activate glycogen synthesis [29]. The expression of GSK3β protein was significantly increased, and pGSK3β/GSK3β ratio was significantly decreased in the model group (*p* < 0.05), consistent with the results of reduced glycogen content in the model group. The expression of GSK3β was increased in each intervention group, and IRP-2 could significantly promote the phosphorylation of GSK3β. Itziar Eseberri et al. illustrated that resveratrol and quercitrin could reduce GSK3β protein expression, increase phosphorylation, and increase glycogen content [30].

GCK is the vital glucose sensor in pancreatic β-cells, controlling insulin secretion and maintaining glucose homeostasis [31]. G6PC and PEPCK are key enzymes of the gluconeogenic pathway, and their abnormal activation promotes the conversion of non-glucose substances into glucose and transports them to the vasculature, leading to hyperglycemia. Nuclear factor Y transcription factor causes the activation of the transcription of gluconeogenic genes by directly binding PEPCK and G6PC [32]. The activity of GCK was significantly decreased, and the activities of G6PC and PEPCK were significantly elevated in the model group (*p* < 0.05). GCK activity in each intervention group was significantly elevated (*p* < 0.05), and the activities of G6PC and PEPCK were significantly decreased (*p* < 0.05). IRP-1 and IRP-4 groups exhibited the most significant effects, and the RP effect was positively correlated with the dose. To verify the enzyme activity index, we measured transcript levels of glucose metabolism genes. Marine da et al. discovered that insulin sensitivity and insulin resistance could be improved by increasing *GCK* gene expression [33]. Linyi Shu et al. revealed that the expression of *G6PC* and *PEPCK* genes was greatly increased in T2DM mice, which regulated the conversion of large amounts of non-sugar substances into glucose, resulting in hyperglycemia [34]. The mRNA level of the *GCK* gene increased significantly in each intervention group. RP-200 and IRP-4 groups increased most markedly. The mRNA levels of *G6PC* and *PEPCK* genes decreased significantly in each intervention group. IRP-3 and IRP-4 groups had the most marked effects. Diana et al. demonstrated that cinnamon polyphenol reduced hepatic gluconeogenesis and decreased *PEPCK* and *G6PC* gene expression [35].

The P13K/AKT signaling pathway is a core downstream molecular pathway of insulin. PI3K catalyzes phosphatidylinositol 4,5-bisphosphate (PIP2) to generate PIP3. PIP3 acts as a second messenger to activate AKT. The activated Akt inhibits the expression of FOXO1 and then promotes glucose uptake and glycogen synthesis, to ameliorate hyperglycemia [36]. AKT is a pivotal factor in the insulin signaling pathway, and p-AKT regulates several key factors, including GSK3β FOXO1, to further regulate glucose and lipid metabolism stability. The results showed that RP and IRP 1–4 could alleviate IR by up-regulating P13K and p-AKT expression, and down-regulating FOXO1 expression in db/db mice.

Nonetheless, the ability of IRP-3 to up-regulate the expression of P13K protein was lower than other IRPs, and the ability of IRP 1–4 to increase the phosphorylation of AKT protein was lower than other IRPs. These results indicate that IRP-1 and IRP-2 were the principal active substances that activated the P13K/AKT/FOXO1 signaling pathway in RP. IRP-4 had a relatively satisfactory hypoglycemic effect, but the primary hypoglycemic mechanism may be a result of other signaling pathways. Heng et al. also found that administration with quercitrin isolated from custard apple leaves could significantly reduce the glucose level in IR cells [37]. Our research further confirms that quercitrin might improve insulin sensitivity through the P13K/AKT pathway.

## 5. Conclusions

In summary, the oral administration of RP and IRP 1–4 markedly ameliorated hyperglycemia in the T2DM mice, characterized by a decrease in the food and water intake, FBG, and enhanced glucose tolerance. RP and IRP 1–4 interventions could significantly ameliorate the IR and improve the activities of CAT and T-SOD in the T2DM mice. Moreover, RP and IRP 1–3 up-regulate the P13K/AKT insulin signaling pathway, further regulating the expression of the p-GSK3β protein and FOXO1 protein, resulting in the promotion of glycogen storage and the inhibition of gluconeogenesis. IRP-4 might ameliorate hyperglycemia through other pathways. The results suggest that RP might be utilized as a hypoglycemic ingredient for improving glucose metabolism disorder.

## Figures and Tables

**Table 1:** Sequences of Primers for Genes.

Gene	Direction	Primer Sequences (5′−3′)
*GCK*	sense	GCTTCACCTTCTCCTTCCCTGTA
	antisense	CACGATGTTGTTCCCTTCTGCT
*G6PC*	sense	CCTGAGGAACGCCTTCTATGTC
	antisense	GAGCTGTTGCTGTAGTAGTCGGT
*PEPCK*	sense	GCAAGACAGTCATCATCACCCA
	antisense	GGCGAGTCTGTCAGTTCAATACC
*GADPH*	sense	CCTCGTCCCGTAGACAAAATG
	antisense	TGAGGTCAATGAAGGGGTCGT

**Table 2:** Effects of RP and IRP 1–4 on oxidative stress in db/db mice of 0 and 8 weeks.

Group	MDA (Serum) (nmol/mL)	T-SOD (Serum) (U/mL)	T-AOC (Liver) (mM)	CAT (Liver) (U/mgprot)	MDA (Liver) (nmol/mgprot)	T-SOD (Liver) (U/mgprot)
Control	7.372 ± 1.049 ^e^	118.025 ± 2.764 ^a^	1.122 ± 0.241 ^a,b^	40.113 ± 2.023 ^b,c,d^	9.370 ± 2.663 ^b^	308.717 ± 16.585 ^a^
Model	16.993 ± 1.445 ^a^	88.198 ± 3.994 ^f^	0.885 ± 0.051 ^c^	32.052 ± 5.156 ^e^	20.446 ± 5.335 ^a^	230.777± 20.503 ^c^
Positive	14.137 ± 0.683 ^b^	99.95 ± 1.387 ^e^	1.02 ± 0.097 ^b,c^	35.623 ± 2.314 ^d,e^	9.361 ± 3.876 ^b^	256.716 ± 25.589 ^b^
RP-100	12.135 ± 0.695 ^c^	104.738 ± 5.347 ^d^	0.878 ± 0.106 ^c^	37.232 ± 4.823 ^d,e^	13.847 ± 5.960 ^b^	247.021 ± 30.589 ^b,c^
RP-200	10.742 ± 0.509 ^d^	111.673 ± 2.886 ^b,c^	1.133 ± 0.319 ^a,b^	45.717 ± 6.786 ^a,b,c^	13.512 ± 2.748 ^b^	264.893 ± 13.923 ^b^
IRP-1	10.083 ± 0.437 ^d^	115.192 ± 2.054 ^a,b^	1.062 ± 0.132 ^a,b^	47.187 ± 3.138 ^a^	9.521 ± 4.473 ^b^	268.55 ± 17.887 ^b^
IRP-2	13.730 ± 0.657 ^b^	111.773 ± 5.117 ^b,c^	1.348 ± 0.316 ^a^	46.678 ± 7.533 ^a,b^	9.767 ± 2.473 ^b^	265.666 ± 28.190 ^b^
IRP-3	14.397 ± 0.671 ^b^	108.062 ± 1.964 ^c,d^	1.062 ± 0.238 ^a,b^	39.447 ± 6.211 ^c,d^	8.495 ± 2.330 ^b^	252.766 ± 7.854 ^b^
IRP-4	10.432 ± 0.975 ^d^	114.802 ± 1.866 ^a,b^	1.240 ± 0.159 ^a,b^	39.500 ± 2.235 ^c,d^	9.428 ± 2.318 ^b^	248.097 ± 11.144 ^b^

All data are expressed as mean ± SD. Different letters (superscripts a–f) are markedly different at *p* < 0.05.

## Data Availability

The data that support the findings of this study are available from the corresponding author upon reasonable request.

## Supplementary Materials

The following supporting information can be downloaded at: https://www.mdpi.com/article/10.3390/foods11050636/s1, Table S1: Preparation of Reverse Transcription Reaction System; Table S2: Reverse Transcription Program Settings; Table S3: the QPCR Teaction System; Table S4: PCR Amplification Procedure; Table S5: The ratio of Separating Gel and Stacking Gel.
