# Supplementary material for: Polyphenol from Rosa roxburghii Tratt Fruit Ameliorates the Symptoms of Diabetes by Activating the P13K/AKT Insulin Pathway in db/db Mice
Source: Foods. 2022 Feb 22;11(5):636. doi: 10.3390/foods11050636 (PMC8909201; doi:10.3390/foods11050636)
Supplement: Supplementary file 1 [file foods-11-00636-s001.zip › foods-1583584-supplementary.pdf]

Table S1. Preparation of Reverse Transcription Reaction System

Component	Volume
5 x Reaction Buffer	4 μL
Oligo $(dT)_{18}$ Primer (100 μM)	0.5 μL
And Random Hexamer primer (100 μM)	0.5 μL
Servicebio®RT Enzyme Mix	1 μL
Total RNA *	10 μL
RNase free water	Add to 20 μL

Table S2. Reverse Transcription Program Settings

Temperature	Time
25℃	5 min
42℃	30 min
85℃	5 sec

Table S3. The QPCR Reaction System

2× qPCR Mix	7.5 μl
2.5μM Gene primers (upstream + downstream)	1.5 μl
reverse transcript（cDNA）	2.0 μl
ddH_2O	4.0 μl

Table S4. PCR Amplification Procedure

Stage1	Stage2（40 loops）	Stage3（melting curve）
95 ℃，10 min predenaturation	95 ℃，15s degeneration	65 ℃→95 ℃
	60 ℃，30 s Annealing/Extension	Every time the temperature rises 0.5 ℃, the fluorescence signal is collected once

Table S5. Ratio of Separating Gel and Stacking Gel

	Separating glue ratio					
Reagents	8 %	10 %	12 %	15 %	18 %	20 %
H_2O (ml)	4.63	4	3.3	2.3	1.3	0.63
30％ Acrylamide（29:1）(ml)	2.67	3.3	4	5	6	6.67
1.5M TRIS－Hcl(PH 8.8) (ml)	2.5	2.5	2.5	2.5	2.5	2.5
10％SDS (ml)	0.1	0.1	0.1	0.1	0.1	0.1
AP (ml)	0.1	0.1	0.1	0.1	0.1	0.1
TEMED (ul)	5 ul	5 ul	5 ul	5 ul	5 ul	5 ul
Total volume (ml)	10 ml					

Reagent	5 % stacking gel ratio
H_2O ml	2
30％ Acrylamide（29:1）(ml)	0.5
1M TRIS－Hcl (PH 6.8) (ml)	0.5
10％SDS (ul)	40
AP (ul)	30
TEMED (ul)	4 ul
Total volume (ml)	3 ml
